# A Critical Examination of the Changes Proposed by the AJCCCCEP for the AJCC 9th Edition Colorectal Cancer Staging System

**DOI:** 10.1097/PAS.0000000000002539

**Published:** 2026-04-10

**Authors:** Angela Yang Pan, Hojabr Kakavand, Loretta Sioson, Amy Sheen, Mahsa S. Ahadi, Angela Chou, Anthony J. Gill

**Affiliations:** * Sydney Medical School, University of Sydney, Sydney; † Cancer Diagnosis and Pathology Group, Kolling Institute of Medical Research; ‡ NSW Health Pathology, Department of Anatomical Pathology, Royal North Shore Hospital, St Leonards, NSW, Australia

**Keywords:** colorectal carcinoma, CRC, AJCC staging, TNM staging, tumor deposits

## Abstract

The AJCC 8th edition colorectal cancer (CRC) staging system demonstrates non-hierarchical survival outcomes, with stage IIIA survivals consistently approximating stage I and exceeding stage II. The AJCC Colon Cancer Expert Panel (AJCCCCEP) has attempted to address this with its new proposal for the 9th edition, which incorporates tumor deposits (TDs) as an independent parameter and rebalances the relative significances of the T-stages and N-stages. We sought to critically assess these proposals in a large single-centre cohort. Patients undergoing CRC resection at our institution from 2005 to 2024 were staged according to the proposed system. Overall survival (OS) was evaluated using Kaplan-Meier analysis and pairwise comparisons made through log-rank tests (*P* < 0.05). Four thousand two hundred fifty-seven CRC patients were identified (median age 74 y, 51% female; 36% node-positive, 17.5% TD positive). Median duration of follow-up was 8.5 years. The proposed staging produced hierarchical OS separation, and all pairwise comparisons achieved statistical significance except stage I versus IIA (*P*=0.5), I versus IIB (*P*=0.081), and IIA versus IIB (*P*=0.10). Surprisingly, in subgroup analysis, patients with low T-stage (T1/T2) with lymph node metastases or tumor deposits (who comprised a small component of the proposed stage IIB grouping) demonstrated superior survival compared with T1/T2 node and tumor deposit-negative patients (*P*=0.014). We conclude that the changes proposed by the AJCCCCEP for the 9th edition are an improvement and achieve hierarchical survival stratification. However, if our findings are confirmed in other independent cohorts, the proposal’s application in T1/T2 stage disease may need refinement.

Accurate pathologic staging of colorectal carcinoma (CRC) guides treatment and predicts outcomes. Inaccurate or inconsistent staging is associated with suboptimal treatment and worse outcomes.^1^ The American Joint Committee on Cancer (AJCC) 8th edition system is the current international standard for staging CRC.^2^ It is based on the key parameters of tumor depth (pT), nodal involvement (pN), and distant metastases (pM).

A significant weakness of the 8th edition AJCC staging system for CRC is that it produces non-hierarchical survival outcomes. Under this edition, stage IIIA patients are those with very low T-stage (T1) and moderate numbers of positive nodes (1 to 6 nodes), or low T-stage disease (T2) and few lymph node metastases (1 to 3 nodes). These 8th edition stage IIIA patients consistently demonstrate similar survival to stage I patients (patients with similar low T-stages and no lymph node metastases); and show better survival than patients with stage IIA, IIB, or IIC disease who have higher T-stage (T3, T4A, or T4B, respectively) but lack lymph node metastases (N0).^3–6^ This finding, which has been termed *the stage IIIA paradox*,^5^ is independent of whether the patients received adjuvant therapy, and is attributable to under-estimating the prognostic significance of deeper invasion into the bowel wall (ie, higher T-stage, especially T3, T4A, or T4B), and over-estimating the significance of small numbers of positive lymph nodes (N1 disease).^5–10^

A second problem with the 8th edition staging system is with its treatment of discontinuous tumor deposits (TDs). Although criteria have changed over the last few decades and the assessment of TDs may be subject to interobserver variance, TDs can be broadly defined as deposits of carcinoma in the soft tissue that are separate from the main tumor mass, not clearly associated with lymph nodes, and are within the lymphatic drainage area of the primary tumor.^5,11^ TDs are common, being found in ~20% of all CRCs.^5,12^ Under the 8th edition staging system, TDs do not alter the stage of patients who have lymph node metastases. In patients without lymph node metastases, TDs warrant classification as nodal group N1C, which is treated identically for staging purposes to N1A or N1B (1 to 3 nodes). This is despite overwhelming evidence that TDs are a highly adverse independent prognostic finding, likely with greater significance than small numbers of lymph node deposits.^5,11–18^

To address these issues, the AJCC Colon Cancer Expert Panel (AJCCCCEP) has proposed changes for the upcoming AJCC 9th edition staging system with the explicit aim of restoring hierarchical survival outcomes by rebalancing the relative significance of T-stage and N-stage, and more accurately reflecting the adverse prognostic significance of TDs.^19^ The proposals are summarized in Table 1.

**TABLE 1:** Comparison of the Current 8th Edition AJCC Staging System for Colorectal Carcinoma With the Proposals for the 9th Edition made by the AJCCCEP Group^19^

AJCC 8^th^ edition				Proposed 9^th^ edition				
Stage	T	N	M	Stage	T	# of LN+	# of TD	M
**0**	Tis	N0	0	**—**	—	—	—	—
**I**	T1-T2	N0	0	**I**	T1	0	0	0
**IIA**	T3	N0	0	**IIA**	T2	0	0	0
**IIB**	T4a	N0	0	**IIB**	T1	0	1+	0
					T1	1+	0	0
					T2	0	1+	0
					T2	1-4	0	0
					T3	0	0	0
**IIC**	T4b	N0	0	**—**	—	—	—	—
**IIIA**	T1-T2T1	N1/N1c N2a	00	**IIIA**	T1	1+	1+	0
					T2	1-4	1+	0
					T2	5+	0	0
					T3	0	1+	0
					T3	1-4	0	0
**IIIB**	T3-T4aT2-T3T1-T2	N1/N1c N2a N2b	000	**IIIB**	T2	5+	1+	0
					T3	1-4	1+	0
					T3	5+	0	0
					T4a	0-4	0	0
					T4b	0-2	0	0
**IIIC**	T4aT3-T4aT4b	N2a N2b N1-N2	000	**IIIC**	T3	5+	1+	0
					T4a	0-4	1+	0
					T4a	5+	Any	0
					T4b	0-2	1+	0
					T4b	3+	Any	0
**IVA**	Any	Any	1a	**IVA**	Any	Any	Any	1a
**IVB**	Any	Any	1b	**IVB**	Any	Any	Any	1b
**IVC**	Any	Any	1c	**—**	—	—	—	—

AJCC indicates American Joint Committee on Cancer; LN+, positive lymph nodes; TD, tumor deposits.

Briefly, under these proposed changes TDs are recognized as a distinct (and highly significant) component of the grading system, separate to N-stage. At the same time the T and N-stages are rebalanced, placing greater weight on T-stage and less weight on N-stage, particularly for patients with low numbers of positive lymph nodes. Stage I and IIA comprise patients with low T-stage (T1 or T2, respectively) and no involved lymph nodes and no TDs. Stage IIB then comprises patients with any of the following: very low T-stage (T1) and any number of involved nodes or TDs (but not nodes and TDs); low T-stage (T2) and low numbers of involved nodes (1 to 4 nodes) or TDs (but not nodes and TDs); or intermediate T-stage (T3) and no positive nodes or TDs. Stage IIC is abolished.

The proposed stage IIIA comprises patients with very low T-stage (T1) and any numbers of lymph nodes as well as having TDs; or patients with low T-stage (T2) and both low numbers of involved nodes (1 to 4) and TDs; or patients with low T-stage (T2) and large numbers of involved nodes (5 or more) but no TDs; or patients with intermediate T-stage (T3) and low numbers of nodes or TDs (but not nodes and TDs). Stage IIIB and IIIC comprise patients with either T3 disease with progressively more nodal metastases and/or TDs, or serosal involvement (T4 disease).

By moving beyond a nodal-centric framework, this new approach better reflects the adverse prognostic impact of more advanced local disease (represented by T-stage) and TDs, while decreasing the impact of small numbers of positive lymph nodes in low T-stage disease. However, these proposals need to be validated in large independent cohorts. We therefore sought to investigate the proposed AJCCCCEP 9th edition staging framework in a large single‑center cohort to determine whether this new system achieves improved hierarchical separation of survival outcomes. We went on to examine whether the new proposals work equally well in different locations, such as the rectum or the right colon versus the left colon. Finally, we examined the relative contributions of T-stage, N-stage, and TDs in different cohorts to determine if there were some specific subgroups (eg, those with low T-stages) for which the new staging system could be further improved.

## METHODS

A retrospective review was conducted using the pathology database of the Department of Anatomical Pathology at Royal North Shore Hospital (Sydney, Australia) to identify all patients who underwent surgical resection for colorectal carcinoma (CRC) between 1 January 2005 and 31 December 2024. Patients were excluded if the primary lesion arose in the vermiform appendix or outside the colon, if management consisted only of biopsy or endoluminal therapy, if distant metastases (stage IV disease) were present, or if histology did not meet the World Health Organization 2020 criteria for adenocarcinoma.^20^ Rectal cancers were included in the study cohort, including those treated with neoadjuvant therapy, provided that a definitive surgical resection was performed. In patients with multiple synchronous primaries, the tumor with the highest pathologic stage was selected for analysis. For binary analysis of tumor site, location was classified into 2 groups: right colon (cecum, ascending colon, hepatic flexure, and transverse colon) versus left colon (splenic flexure, descending colon, sigmoid, and rectum). Separately, the performance of the staging system in rectal carcinomas was assessed.

From 2005 onwards, the surgical pathology of colorectal resections at our institution has been reported using structured, synoptic templates designed to capture all major pathologic variables, including the identification of TDs. However, the precise criteria used to define TDs pathologically and separate them from lymph node metastases or foci of extramural venous invasion [EMVI] and perineural invasion [PNI] have changed over the 20-year period of data collection. Fortunately, our structured reporting template always included a line item for deposits of tumor in soft tissue away from the leading edge of the tumor not clearly within lymph nodes. Although we did not review all the cases, this approach would capture all tumors with potential TDs; and if there was any doubt whether tumors previously considered positive for TDs would be considered positive by current criteria (having considered how our criteria at the time of reporting during different time periods differed from the new consensus criteria), we were then able to review the original slides.

For the purposes of this study, TDs are defined using the criteria recently proposed by Nagtegaal et al^21^ which were developed after international consensus and are intended to be the criteria used for the 9th edition. Under this definition, TDs are defined as follows:^21^

*TDs represent discrete tumor nodules of any shape, contour, or size in perirectal and pericolonic fat away from the leading edge of the tumor within the lymph drainage area of the primary carcinoma. TD can originate from different histologic structures, including lymph nodes, vessels, and nerves. Therefore, TD may contain foci of extramural venous invasion EMVI and PNI. The feature distinguishing a TD from EMVI and PNI is the presence of unequivocal tumor extension from the vessel or nerve into the surrounding fat or fibroconnective tissue. When tumor outgrowth from EMVI and/or PNI is present, the diagnosis of TD and EMVI/PNI should be denoted separately in the report. If the tumor involves an identifiable lymph node, it is considered as lymph node metastasis and not as TD even if the tumor extends into the perinodal fat.*

**FIGURE 1:**
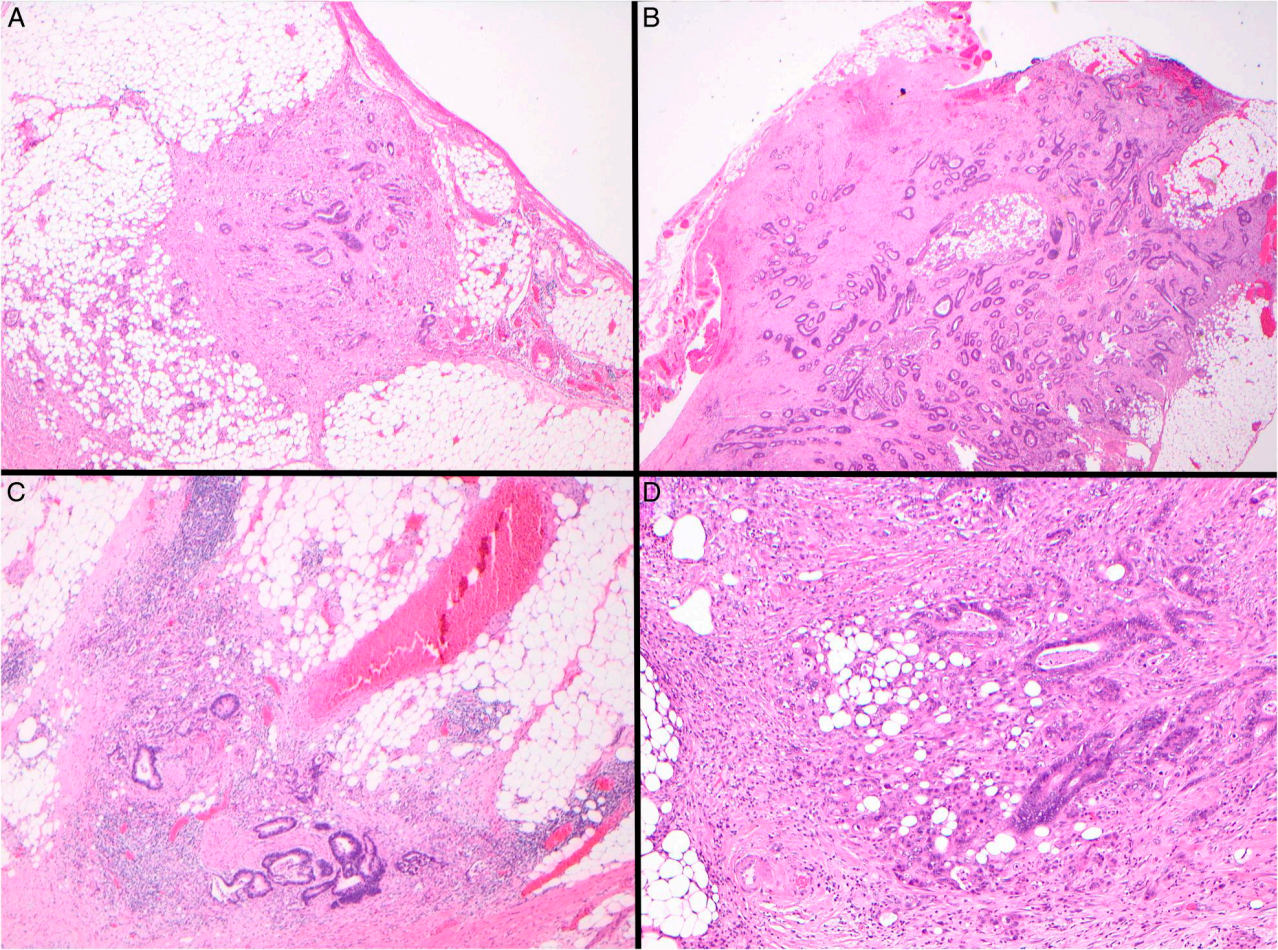
A and B, Tumor deposits (TDs) are defined as discrete tumor nodules of any shape, contour, or size in perirectal and pericolonic fat away from the leading edge of the tumor within the lymph drainage area of the primary carcinoma. C and D, Although tumor deposits may arise from perineural growth or extramural venous invasion, there must be unequivocal tumor extension from the vessel or nerve into the surrounding connective tissue and the tumor focus should be larger than the nerve.

It is beyond the scope of this publication to detail all the specific criteria proposed for the 9th edition, and we refer the reader to Nagtegaal et al’s^21^ report for more detail. However, we accept that many cases may be prone to interobserver discrepancy, and we do highlight a few specific situations where the new consensus criteria may help to improve concordance based on which cases we felt prudent to review. Specifically, there is now no size cutoff for TDs as this would be considered arbitrary, but in the specific situation described above of distinguishing TDs from PNI, the new consensus criteria recommend that the tumor focus should be *larger than the nerve* (as well as extend into the adjacent fat and fibroconnective tissue).^21^ Similarly for the specific question of how much separation there should be between a TD and the invasive front of the tumor for a TD to be considered discontinuous, it was agreed under the new consensus criteria that *there is no minimum distance requirement if the tumor focus is not continuous with the primary tumor.* ^21^ Examples of TDs are presented in Figure 1.

All tumors were staged according to the AJCC 8th edition and subsequently re-staged under the proposed AJCCCCEP 9th edition staging framework as outlined in Table 1.^19^ Overall survival (OS) was defined as the interval from surgery to death from any cause, with follow-up censored on June 30, 2025. Kaplan-Meier OS curves were generated for both the AJCC 8th edition and the proposed AJCCCCEP 9th edition staging framework, stratified by the stages defined by each system. Categories with n<10 were excluded from survival curves due to small sample size. To quantify the relative risk of death between stages, Cox proportional hazards models were fitted with stage as a categorical predictor. Stage I was used as the reference category. Pairwise comparisons between stages were performed using estimated marginal means derived from the Cox models, and hazard ratios (HRs) with 95% CIs were reported. To account for multiple testing, *P*-values from pairwise comparisons were adjusted using the Holm method. Hazard ratios <1 indicate lower mortality risk for the first-listed stage relative to the comparator stage. Statistical significance was set at *P*<0.05. Subgroup analyses compared overall survival (OS) across stages between right and left colon under the proposed 9th edition staging system. Survival outcomes were also stratified by T-stage and nodal involvement. All statistical analyses were performed using RStudio (v2025.09.0+387). This study was approved by the NSLHD ethics committee: ref 2019/ETH08420.

## RESULTS

### Cohort Characteristics

A total of 4257 patients with CRC undergoing surgical resection between 2005 and 2024 were identified and met inclusion criteria. The clinical and pathologic details of the cohort are presented in supplementary table 1, Supplemental Digital Content 1, http://links.lww.com/PAS/C268. The median age at surgery was 74 years (interquartile range [IQR]: 64 to 82 y); 51% were female. By 8th edition TMN staging, 876 patients (21%) were stage I, 1690 (40%) were stage II, and 1691 (40%) were stage III. Tumor deposits were present in 743 cases (17%) and absent in 3514 (83%). Lymph node involvement was found in 1539 (36%) and absent in the remaining 2718 patients (64%).

### Survival Analysis

**FIGURE 2:**
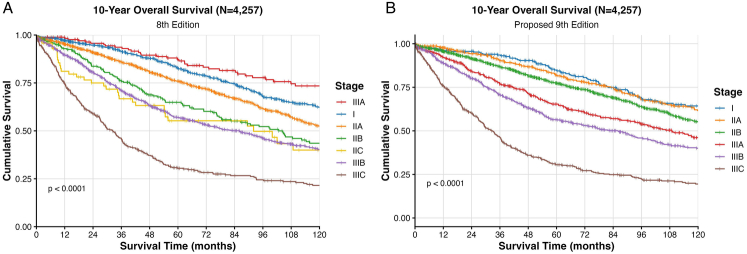
Overall survival according for the cohort when stratified by the AJCC (A) 8th and (B) proposed 9th edition staging systems (N=4257).

The median duration of follow-up was 8.5 years, and the estimated median overall survival for the entire cohort was 113 months. The Kaplan-Meier survival curves when stratified by both the AJCC 8th edition and proposed 9th edition are presented in Figure 2.

Under AJCC 8th edition staging (Fig. 2A), stage was significantly associated with overall survival (global log-rank *P*<0.001). However, as expected, the survival outcomes were not hierarchical. In fact, the 5-year overall survival was highest for stage IIIA (87.3%, 95% CI: 82.2-92.9%), exceeding all other stages including stage I (82.7%), stage IIA (76.0%), stage IIB (64.9%), and stage IIC (55.3%). Similarly, overall survival censored at 10 years for stage IIIA (73.5%, 95% CI: 65.9-81.8%) exceeded that observed in stage I (62.2%), stage IIA (52.5%), stage IIB (43.5%), and stage IIC (40.0%). Compared with stage I, stage IIIA was associated with a lower mortality risk (HR: 0.72, 95% CI: 0.53-0.97); however, this difference was not statistically significant (*P*=0.13). Detailed pairwise comparisons are presented in supplementary table 2, Supplemental Digital Content 2, http://links.lww.com/PAS/C269. In addition to those attributable to the stage IIIA paradox, there were other survival curves that failed to separate. For example, there was no statistically significant difference in survival for IIB versus IIC (HR: 1.27 95% CI: 0.72-2.22, *P*=0.58), IIB versus IIIB (HR: 1.14, 95% CI: 0.84-1.54, *P*=0.58), and IIC versus IIIB (HR: 0.90, 95% CI: 0.54-1.49, *P*=0.58). However, all other stage groups showed statistically significant separation in pairwise comparisons including stage I versus IIA (HR: 1.28, 95% CI: 1.02-1.61, *P*=0.006), stage IIA versus IIB (HR: 1.35, 95% CI: 0.99-1.82, *P*=0.015), and stage IIIB versus IIIC (HR: 1.92, 95% CI: 1.54-2.38, *P*<0.001).

In contrast, staging by the proposed 9th edition (Fig. 2B) produced hierarchical separation of survival curves across all stages (global log-rank *P*<0.0001), at both 5 and 10 years of follow-up. Detailed pairwise comparisons are presented in supplementary table 2, Supplemental Digital Content 2, http://links.lww.com/PAS/C269. Although stage I patients demonstrated the best survival, stage I survival was not significantly different from stage IIA patients (HR: 1.09, 95% CI: 0.75-1.59; *P*=0.50) and just failed to demonstrate statistical significance with stage IIB patients (HR: 1.28, 95% CI: 0.92-1.79; *P*=0.081). Although survivals for stage IIA versus IIB (HR: 1.18, 95% CI: 0.92-1.52, *P*=0.10) also just failed to demonstrate statistical significance, all other stage groups showed statistically significant separation in pairwise comparisons.

### Right Versus Left Colon, and Rectal Tumors

**FIGURE 3:**
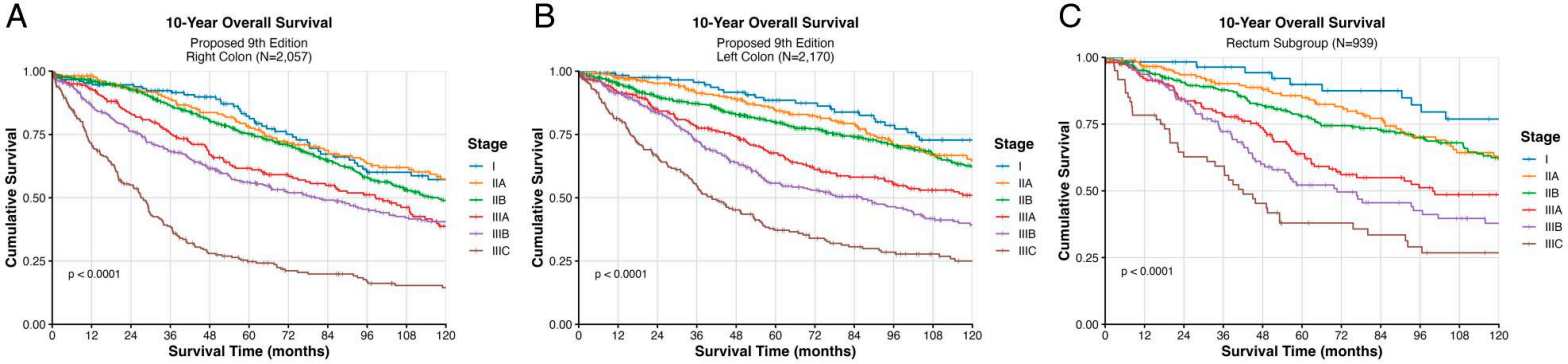
Under the 9th edition proposals, the survival curves remained hierarchical under subgroup analysis for right (A) and left (B) sided tumors; as well as for (C) rectal tumors.

Having demonstrated the utility of the proposed 9th edition staging system in the entire cohort, we then sought to investigate whether the new staging system proposal worked equally well for right and left-sided tumors and for rectal tumors. Again, as demonstrated in Figure 3, the survival curves for both right-sided and left-sided, as well as rectal tumors remained hierarchical and statistically significant (*P*<0.0001).

### Relative Contribution of T-Stage and Nodal Status

**FIGURE 4:**
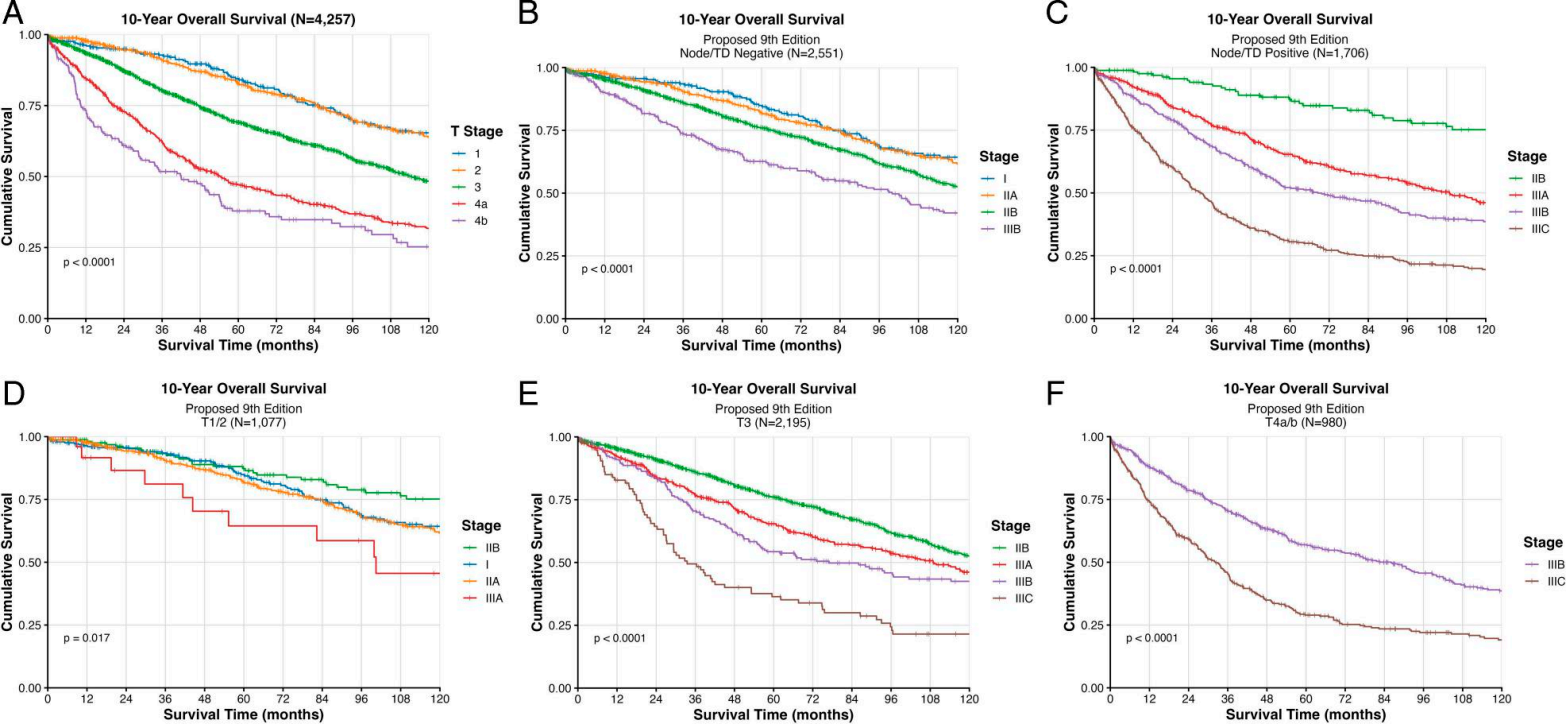
Overall survival curves stratified by different parameters using the AJCCCCEP 9th edition proposals. A, When stratified by T-stage, there was no significant difference T1 versus T2, but other curves separated. B, In the subgroup of node/TD-negative patients there was no separation of stage I and IIA, but IIB (stage T3) and IIIB (stage T4a/b) separated. C, In the subgroup of node-positive patients all 4 curves separated. D, When exclusively low T-stage (T1/T2) patients were considered, paradoxically stage IIB patients (T1 patients with any number of node metastases or TDs; and T2 patients with low numbers of node metastases and TDs) demonstrated improved survival compared with stage I and IIA patients (T1/T2 patients who were node negative). In contrast, the survival curves of patients with T3 (E) and T4 (F) separated when stratified by the proposed nodal/TD groups.

We then performed subgroup analysis to determine the relative contribution of T-stage and nodal/TD status to the 9th edition proposals, specifically to investigate whether there are any specific combinations for which survival is non-hierarchical. Survival curves were first created based on T-stage alone (Fig. 4A). The survival in T1 and T2 patients was similar (HR T2 vs. T1: 1.03, 95% CI: 0.70-1.35, *P*=0.81), followed by progressively lower survival in T3 (HR vs. T1: 1.61, 95% CI: 1.21-2.15, *P*<0.0001). Survival was lowest in the T4 group, with T4a (HR vs T1: 2.77, 95% CI: 2.05-3.76, *P*<0.0001) and T4b (HR vs T1: 3.74, 95% CI: 2.56-5.46, *P*<0.0001). T4b had higher risk than T4a (HR: 1.35, 95% CI: 1.01-1.81, *p* 0.0076). In the subgroup of node/TD-negative patients, we then examined the separation of the survival curves (Fig. 4B). As expected, there was no separation for stage I and IIA (as these represent T1 and T2 node-negative tumors only), but there was separation of IIB (T3) and IIIB (T4) patients (*P*<0.0001). When the subgroup of all node-positive patients was examined (Fig. 4C), there was good separation of all 4 groups—IIB, IIIA, IIIB, and IIIC (*P*<0.0001).

Noting that patients with T1 and T2 disease without lymph node involvement (proposed 9th edition stage I and IIA) demonstrated similar survival, we sought to investigate whether the way N-stage and TDs were integrated with T1/T2 patients in the 9th edition was appropriate. We therefore created survival curves for patients with T1/T2 disease, who have varying combinations of lymph nodes and TDs, stratified under the 9th edition proposals (Fig. 4D). Surprisingly, in the low T-stage patients (T1/T2), those with stage IIB disease (that is those patients with T1 and any number of nodal metastases or TDs, or T2 disease and either small numbers of lymph node metastasis or TDs) demonstrated better survival than patients with stage I or IIA disease (that is T1/T2 patients without lymph node metastases). In contrast, when we stratified T3 (Fig. 4E) and T4 (Fig. 4F) patients by the proposed 9th edition groupings, all the survival curves remained hierarchical.

**FIGURE 5:**
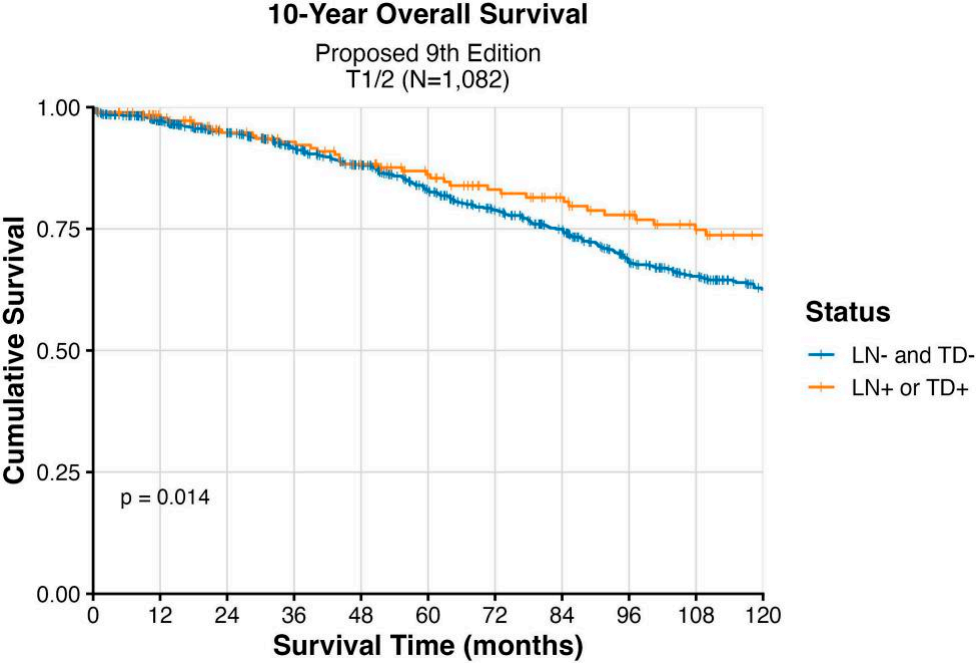
To investigate why patients with the proposed stage IIB demonstrated better survival than patients with stage I/IIA disease, we stratified T1/T2 patients by the presence of lymph node metastases and/or TDs and found that these low T-stage patients with node metastases or TDs actually demonstrated better survival than node/TD-negative patients.

To further investigate the surprising finding that patients with low T-stage (T1/T2) but overall stage IIB disease demonstrated better survival than stage I/IIA patients, we stratified patients with T1/T2 disease by the presence or absence of any number of involved nodes and/or TDs (Fig. 5). This confirmed that, in the setting of low T-stage (T1/T2) disease, patients with any number of positive lymph nodes and/or TDs demonstrated better survival than patients with no involved nodes or TDs (*P*=0.014).

**FIGURE 6:**
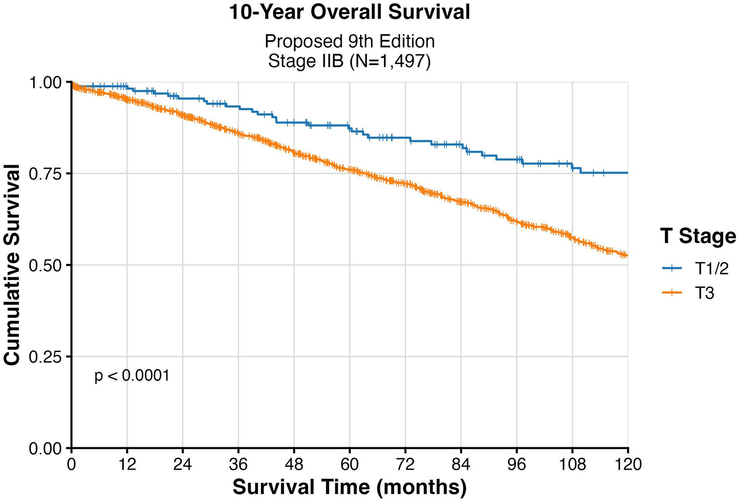
To further investigate why patients with proposed stage IIB demonstrated better survival than patients with stage I/IIA disease, we stratified stage IIB patients by those with stage T3 disease (who, by definition, were node/TD-negative) from T1/T2 patients (who, by definition, had either node metastases or TDs). The T3 patients demonstrated significantly worse survival (*P*<0.0001).

Stage IIB under the 9th edition proposals includes both T1/T2 patients with low number of lymph nodes or TDs, and T3 patients with no nodes or TDs. We postulated that the reason survival appeared hierarchical in all stage IIB patients, but not in those with T1/T2 disease is that T3 patients with no nodal metastasis (who actually constitute the great majority of IIB patients, N=1327) do significantly worse than T1/T2 patients with low numbers of nodes or TDs (who are relatively rarer (N=170) and therefore do not determine the overall prognosis of the proposed IIB group). We therefore examined the cohort of all proposed 9th edition IIB patients and divided them into T1/T2 and T3 (Fig. 6). Using this approach, within the IIB grouping, the T3 patients demonstrated significantly worse survival than the T1/T2 patients (*P*<0.0001).

## DISCUSSION

This study is the first evaluation of the proposed AJCCCCEP 9th edition staging system for colorectal cancer in a large independent surgical cohort. Compared with the AJCC 8th edition, which produced non-hierarchical survival outcomes, the 9th edition demonstrated improved stratification of overall survival. Most importantly, the revised system resolves the stage IIIA survival paradox observed under the 8th edition staging system. Although well-known to experts in the field, resolving this paradox is clinically important as non-hierarchical staging increases the difficulty of prognostic counseling and may lead to inappropriate adjuvant treatment decisions.

Right and left-sided colon cancers are both biologically and anatomically different. Right-sided tumors more frequently exhibit microsatellite instability, CpG island methylator phenotype, and immune-rich molecular subtypes, whereas left-sided cancers are typically characterized by chromosomal instability and *TP53*-driven oncogenesis.^22,23^ Furthermore, right-sided cancers are more often diagnosed at older age, are poorly differentiated, and have worse survival outcomes, as demonstrated by large observational studies and meta-analyses.^24–26^ In this context, it is particularly reassuring that the 9th edition proposals maintain hierarchical separation across right colon and left colon, as well as in rectal carcinomas (Fig. 3).

One of the key strengths of the 9th edition proposals is its inclusion of TDs as an adverse prognostic factor, which is independent of nodal status. Although TDs have long been established as a prognostic indicator in colorectal cancer,^5,11–18^ it has been difficult to find a simple approach to integrate them into overall staging—summarized in.^5^ By considering TDs to be a separate parameter to lymph nodes (rather than part of the N-stage), the 9th edition proposals allow the poor prognosis of patients who have both positive nodes and TDs to be considered in the overall stage grouping. In addition to being supported by our empirical data, this approach is biologically plausible, as TDs exhibit aggressive features distinct from both primary tumors and lymph node metastases, including enhanced epithelial-mesenchymal transition and immune evasion.^16^

Although the 9th edition proposals are a significant improvement on the 8th edition, our data does not support all the changes. For example, under the 8th edition, node/TD-negative patients with low stage (T1/T2) were grouped together as stage I. However, under the 9th edition proposals node/TD-negative T1 patients (stage I) are separated from node/TD-negative T2 patients (stage IIA) despite there being no difference in survival (HR: 1.09, 95% CI: 0.75-1.59, *P*=0.50). Although there was a trend towards a survival difference between I and IIA disease and this was a little more prominent in the rectum (Fig. 4C), we can see an argument for not separating node/TD-negative T1 and T2 patients or perhaps making these stage IA and stage IB, respectively, to emphasize the similarities rather than the differences in their outcome.

A second criticism is that stage IIB as defined under the 9th edition proposals represents a heterogeneous group with 2 distinctly different outcomes (*P*<0.0001), comprising those with an intermediate survival (the T3 node/TD-negative patients) and those with an excellent survival (the T1/T2 node/TD-positive patients)—Figures 4D, 5, and 6. It is surprising, and perhaps counterintuitive, that patients with T1/T2 disease with any number of lymph node metastasis or TDs demonstrate significantly better survival than patients with the same T-stage who are node/TD-negative (*P*=0.014)—Figure 5; and we would like to see our data confirmed in other cohorts before changes are suggested to stage groupings. However, although the numbers of patients are small, similar findings have been reported by others,^27^ and there are several plausible reasons why lymph node involvement could be associated with improved survival in low T-stage but not high T-stage (T3/T4) disease. For example, perhaps lymph node metastases/TDs could prime the tumor-specific immune response or perhaps these patients are more likely to receive adjuvant chemotherapy (we do not have access to data on who received adjuvant therapy). In any case, if our findings are confirmed by other groups, it may be appropriate to split the currently proposed IIB group into the good prognosis T1/T2 and the intermediate prognosis T3 cohorts. In fact, depending on what other studies find, it may even be appropriate that any T1/T2 patients be considered low-stage disease regardless of nodal/TD status. Otherwise, the stage IIIA paradox of the 8th edition, may potentially be replaced by the *low T1/T2 stage IIB paradox* in the 9th edition.

The strengths of this study include access to a large surgical cohort (N=4257) with strong linkage to long-term survival data and our alignment with the consensus diagnostic criteria for TDs, which have been proposed for the 9th edition.^21^ However, some limitations should be acknowledged. Firstly, we did not have access to data on which patients received adjuvant chemotherapy and this could confuse interpretation of some cohorts. For example, in the troublesome proposed IIB group, it could be that certain low-risk patients (eg, T1/T2 with positive nodes) may have received adjuvant therapy, whereas some high-risk patients (T3 node negative) may not have received therapy. These are important issues to clarify if withholding adjuvant chemotherapy is to be considered in these low-risk IIB patients based on this data. Secondly, we had inconsistent data on which patients received neoadjuvant therapy. It is reassuring that the proposed staging system still stratified the only subgroup who consistently receive neoadjuvant therapy in our unit—the rectal patients (Fig. 3C). However, it would be beneficial if we had sufficient data and numbers to perform subgroup analysis on the neoadjuvant cohort. Thirdly, overall survival (OS) was used as the primary endpoint as disease-free survival (DFS) and recurrence data were not available. Although OS is the strongest and least subjective of all endpoints, inclusion of DFS and recurrence endpoints in future studies may better capture the biological impact of tumor deposits and early metastatic risk. Fourthly, we analyzed TDs as a binary variable (present vs. absent). Emerging evidence suggests that there is a correlation between the number of TDs and worsening prognosis^28–30^ and in future studies it would be beneficial to record the number of TDs to further refine risk stratification.

Finally, in this study we chose to solely examine staging parameters and not to assess other prognostic factors. In particular, there has recently been considerable interest in tumor budding as a prognostic factor in colorectal carcinoma—summarized in;^31^ and it would be interesting in the future to postulate whether tumor budding could be included in future staging or integrated risk classification schemes. Indeed, going forward it would be beneficial to consider the new staging proposals in the context of budding and multiple other prognostic factors including, but not limited to, grade, vascular space invasion, extramural venous invasion, mismatch repair status, molecular assessment, tumor infiltrating lymphocytes, and perineural growth.

We accept that, despite more explicit consensus definitions, the assessment of TDs is likely to remain subject to interobserver variation. Nevertheless, this study provides the first large-scale investigation of the proposed AJCC 9th edition colorectal cancer staging system in an independent cohort. Our findings are supportive of the proposed framework and confirm that it achieves hierarchical survival outcomes across all colorectal carcinoma patients as well as in specific anatomic cohorts (right colon, left colon, rectum). The observed heterogeneity of the proposed stage IIB cohort and limitations in early T-stage disease highlight areas for further refinement and underscore the need for external validation. Future studies incorporating multi-institutional cohorts, detailed TD quantification, and disease-free survival endpoints will be essential to confirm these findings and inform ongoing optimization of colorectal cancer staging.

## Supplementary Material
