# Supplementary material for: A Critical Examination of the Changes Proposed by the AJCCCCEP for the AJCC 9th Edition Colorectal Cancer Staging System
Source: Am J Surg Pathol. 2026 Apr 10;50(7):770–8. doi: 10.1097/PAS.0000000000002539 (PMC13263040; doi:10.1097/PAS.0000000000002539)
Supplement: Supplementary file 1 [file pas-50-770-s001.docx]

**Supplementary Table 1**

Clinical and pathological characteristics of patients undergoing surgery for colorectal carcinoma from 2005 – 2024 (N=4,257).

|  |  |  | **Univariate analysis** | **Multivariate analysis** |  |  |  |  |
| --- | --- | --- | --- | --- | --- | --- | --- | --- |
| **Variable** | **n (%)** | **Median survival (months)** | **HR** | **95% CI** | ***p*** | **HR** | **95% CI** | ***p*** |
| **Age (years)** |  |  |  |  |  | 1.06 | 1.06-1.07 | **<0.001** |
| >74 | 2,005 (47%) | 196.5 | 1 |  |  |  |  |  |
| ≤74 | 2,252 (53%) | 74.4 | 2.78 | 2.56–3.13 | **<0.001** |  |  |  |
| **Sex** |  |  |  |  |  |  |  |  |
| Female | 1,987 (51%) | 114.2 | 1 |  |  | 1 |  |  |
| Male | 1,907 (49%) | 112.0 | 1.04 | 0.95–1.14 | 0.407 | 1.28 | 1.16-1.4 | **<0.001** |
| Unknown | 363 |  |  |  |  |  |  |  |
| **AJCC stage**  **(8^th^ edition)** |  |  |  |  |  | NI |  |  |
| I | 876 (21%) | 156.4 | 1 |  |  |  |  |  |
| IIA | 1,348 (32%) | 126.5 | 1.28 | 1.11–1.48 | **<0.001** |  |  |  |
| IIB | 270 (6.3%) | 102.7 | 1.72 | 1.39–2.13 | **<0.001** |  |  |  |
| IIC | 72 (1.7%) | 92.1 | 2.18 | 1.55–3.06 | **<0.001** |  |  |  |
| IIIA | 183 (4.3%) | 192.6 | 0.72 | 0.53–0.97 | **0.032** |  |  |  |
| IIIB | 1,070 (25%) | 86.1 | 1.96 | 1.7–2.27 | **<0.001** |  |  |  |
| IIIC | 438 (10%) | 32.3 | 3.74 | 3.17–4.41 | **<0.001** |  |  |  |
| **AJCC stage**  **(9^th^ edition)** |  |  |  |  |  | NI |  |  |
| I | 313 (7.4%) | 168.1 | 1 |  |  |  |  |  |
| IIA | 566 (13%) | 155.4 | 1.09 | 0.85–1.4 | 0.504 |  |  |  |
| IIB | 1,497 (35%) | 136.2 | 1.28 | 1.03–1.6 | **0.027** |  |  |  |
| IIIA | 559 (13%) | 108.9 | 1.68 | 1.32–2.13 | **<0.001** |  |  |  |
| IIIB | 815 (19%) | 86.1 | 2.18 | 1.74–2.73 | **<0.001** |  |  |  |
| IIIC | 507 (12%) | 32.8 | 4.19 | 3.33–5.27 | **<0.001** |  |  |  |
| **Grade** |  |  |  |  |  |  |  |  |
| Low | 3,165 (75%) | 125.1 | 1 |  |  | 1 |  |  |
| High | 1,053 (25%) | 77.9 | 1.53 | 1.38–1.7 | **<0.001** | 1.17 | 1.05-1.31 | **0.004** |
| Unknown | 39 |  |  |  |  |  |  |  |
| **T stage** |  |  |  |  |  |  |  |  |
| 1 | 361 (8.5%) | 164.0 | 1 |  |  | 1 |  |  |
| 2 | 721 (17%) | 165.3 | 1.03 | 0.82–1.3 | 0.806 | 1.07 | 0.85-1.36 | 0.552 |
| 3 | 2,195 (52%) | 114.4 | 1.61 | 1.32–1.97 | **<0.001** | 1.30 | 1.06-1.61 | **0.012** |
| 4a | 791 (19%) | 54.5 | 2.77 | 2.24–3.43 | **<0.001** | 2.06 | 1.65-2.58 | **<0.001** |
| 4b | 189 (4.4%) | 42.0 | 3.75 | 2.87–4.88 | **<0.001** | 2.67 | 2.02-3.53 | **<0.001** |
| **Lymph node involvement** |  |  |  |  |  |  |  |  |
| Absent | 2,718 (64%) | 129.6 | 1 |  |  | 1 |  |  |
| Present | 1,539 (36%) | 72.0 | 1.61 | 1.47–1.76 | **<0.001** | 1.27 | 1.14-1.42 | **<0.001** |
| **Apical node involvement** |  |  |  |  |  |  |  |  |
| Absent | 4,011 (94%) | 119.7 | 1 |  |  | 1 |  |  |
| Present | 236 (5.6%) | 28.9 | 2.77 | 2.36–3.25 | **<0.001** | 2.06 | 1.73-2.46 | **<0.001** |
| **Tumour deposits (TDs)** |  |  |  |  |  |  |  |  |
| Absent | 3,514 (83%) | 128.2 | 1 |  |  | 1 |  |  |
| Present | 743 (17%) | 47.1 | 2.14 | 1.92–2.38 | **<0.001** | 1.67 | 1.47-1.89 | **<0.001** |
| **Site** |  |  |  |  |  | NI |  |  |
| Caecum | 782 (19%) | 87.2 | 1 |  |  |  |  |  |
| Ascending colon | 749 (18%) | 103.4 | 0.90 | 0.78–1.04 | 0.136 |  |  |  |
| Hepatic flexure | 97 (2.3%) | 86.5 | 1.02 | 0.75–1.39 | 0.874 |  |  |  |
| Transverse colon | 429 (10%) | 103.8 | 0.95 | 0.8–1.12 | 0.512 |  |  |  |
| Splenic flexure | 113 (2.7%) | 88.0 | 0.98 | 0.73–1.32 | 0.911 |  |  |  |
| Descending colon | 176 (4.2%) | 108.4 | 0.88 | 0.69–1.12 | 0.298 |  |  |  |
| Sigmoid colon | 942 (22%) | 129.7 | 0.72 | 0.62–0.83 | **<0.001** |  |  |  |
| Rectum | 939 (22%) | 146.7 | 0.67 | 0.58–0.77 | **<0.001** |  |  |  |
| Unknown | 30 |  |  |  |  |  |  |  |
| **Colon site** |  |  |  |  |  |  |  |  |
| Right colon | 2,057 (49%) | 96.4 | 1 |  |  | 1 |  |  |
| Left colon | 2,170 (51%) | 130.9 | 0.76 | 0.69–0.83 | **<0.001** | 0.97 | 0.88-1.08 | 0.613 |
| Unknown | 30 |  |  |  |  |  |  |  |
| **Neoadjuvant therapy** |  |  |  |  |  |  |  |  |
| No/Unknown | 4,169 (97.9%) | 114.3 | 1 |  |  | 1 |  |  |
| Yes | 88 (2.1%) | 52.3 | 1.74 | 1.25–2.43 | **0.001** | 3.19 | 2.24-4.54 | **<0.001** |

Bold *p* values are statistically significant. CI, confidence interval; HR, hazard ratio.; NI, not included (variables excluded from multivariate model due to collinearity or redundancy). *a* Median survival calculated using Kaplan–Meier method. *b* Cox regression model.
