# Supplementary material for: A Critical Examination of the Changes Proposed by the AJCCCCEP for the AJCC 9th Edition Colorectal Cancer Staging System
Source: Am J Surg Pathol. 2026 Apr 10;50(7):770–8. doi: 10.1097/PAS.0000000000002539 (PMC13263040; doi:10.1097/PAS.0000000000002539)
Supplement: Supplementary file 2 [file pas-50-770-s002.docx]

**Supplementary Table 2**

Pairwise hazard ratios for overall survival between AJCC 8^th^ Edition and proposed 9^th^ edition stages

| **Staging System** | **Comparison** | **Hazard ratio (HR)** | **95% CI** | **Adjusted p-value** |
| --- | --- | --- | --- | --- |
| AJCC 8^th^ Edition | I vs IIA | 1.28 | 1.02–1.61 | **0.006** |
|  | I vs IIB | 1.72 | 1.23–2.38 | **<0.001** |
|  | I vs IIC | 2.17 | 1.28–3.70 | **<0.001** |
|  | I vs IIIA | 0.72 | 0.45–1.15 | 0.13 |
|  | I vs IIIB | 1.96 | 1.56–2.44 | **<0.001** |
|  | I vs IIIC | 3.70 | 2.86–4.76 | **<0.001** |
|  | IIA vs IIB | 1.35 | 0.99–1.82 | **0.015** |
|  | IIA vs IIC | 1.69 | 1.02–2.86 | **0.011** |
|  | IIA vs IIIA | 0.56 | 0.36–0.88 | **0.001** |
|  | IIA vs IIIB | 1.54 | 1.27–1.85 | **<0.001** |
|  | IIA vs IIIC | 2.94 | 2.33–3.70 | **<0.001** |
|  | IIB vs IIC | 1.27 | 0.72–2.22 | 0.58 |
|  | IIB vs IIIA | 0.42 | 0.25–0.70 | **<0.001** |
|  | IIB vs IIIB | 1.14 | 0.84–1.54 | 0.58 |
|  | IIB vs IIIC | 2.17 | 1.56–3.02 | **<0.001** |
|  | IIC vs IIIA | 0.33 | 0.17–0.64 | **<0.001** |
|  | IIC vs IIIB | 0.90 | 0.54–1.49 | 0.58 |
|  | IIC vs IIIC | 1.72 | 1.01–2.86 | **0.011** |
|  | IIIA vs IIIB | 2.73 | 1.72–4.35 | **<0.001** |
|  | IIIA vs IIIC | 5.21 | 3.23–8.33 | **<0.001** |
|  | IIIB vs IIIC | 1.92 | 1.54–2.38 | **<0.001** |
| Proposed 9^th^ Edition | I vs IIA | 1.09 | 0.75–1.59 | 0.50 |
|  | I vs IIB | 1.28 | 0.92–1.79 | 0.081 |
|  | I vs IIIA | 1.67 | 1.18–2.38 | **0.0001** |
|  | I vs IIIB | 2.17 | 1.56–3.03 | **<0.001** |
|  | I vs IIIC | 4.17 | 2.94–5.88 | **<0.001** |
|  | IIA vs IIB | 1.18 | 0.92–1.52 | 0.10 |
|  | IIA vs IIIA | 1.54 | 1.16–2.04 | **<0.001** |
|  | IIA vs IIIB | 2.00 | 1.54–2.56 | **<0.001** |
|  | IIA vs IIIC | 3.85 | 2.94–5.00 | **<0.001** |
|  | IIB vs IIIA | 1.32 | 1.04–1.64 | **0.002** |
|  | IIB vs IIIB | 1.69 | 1.41–2.04 | **<0.001** |
|  | IIB vs IIIC | 3.23 | 2.63–4.00 | **<0.001** |
|  | IIIA vs IIIB | 1.30 | 1.03–1.64 | **0.004** |
|  | IIIA vs IIIC | 2.50 | 1.96–3.23 | **<0.001** |
|  | IIIB vs IIIC | 1.92 | 1.56–2.03 | **<0.001** |

Hazard ratios were estimated using Cox proportional hazards models. P-values were adjusted for multiple comparisons using the Holm method. Hazard ratio >1 indicates increased risk of death in the second stage relative to the first stage. Bold p-values are statistically significant.
